# Tool heads prime saccades

**DOI:** 10.1038/s41598-021-91254-8

**Published:** 2021-06-07

**Authors:** Artur Pilacinski, Stella De Haan, Rita Donato, Jorge Almeida

**Affiliations:** 1 Proaction Laboratory, Faculty of Psychology and Educational Sciences, University of Coimbra, 3000-115 Coimbra, Portugal; 2 CINEICC, Faculty of Psychology and Educational Sciences, University of Coimbra, Coimbra, Portugal; 3 GIGA-CRC In Vivo Imaging, University of Liège, Liège, Belgium; 4 Department of General Psychology, University of Padova, Padova, Italy; 5 Human Inspired Technology Research Centre, University of Padova, Padova, Italy

**Keywords:** Human behaviour, Cognitive neuroscience, Motor control

## Abstract

Tools are wielded by their handles, but a lot of information about their function comes from their heads (the action-ends). Here we investigated whether eye saccadic movements are primed by tool handles, or whether they are primed by tool heads. We measured human saccadic reaction times while subjects were performing an attentional task. We found that saccades were executed quicker when performed to the side congruent with the tool head, even though “toolness” was irrelevant for the task. Our results show that heads are automatically processed by the visual system to orient eye movements, indicating that eyes are attracted by functional parts of manipulable objects and by the characteristic information these parts convey.

## Introduction

A typical hand-held tool consists of a handle (the graspable part) and a head (the end through which a tool interacts with the environment; the action-end). Knowing where to grasp a tool is important for holding and using it. Knowing how to use a tool requires recognizing it and its basic function (e.g.^1–3^). These two aspects—how to hold the tool and what the tool does—are crucial for recognizing how we can use it to interact with the environment^4,5^.

While the handle is vital for grasping a tool, the head may bring information about a tool’s actual function and identity, as they may be individualizing properties of each object. Thus, one could expect that the eye may be attracted towards the tool’s head in order to recognize the tool’s distinct characteristics that are used for appropriate grasping in line with tool’s function. The available oculomotor data on tool perception is, however, somewhat inconclusive at showing the influence of tool structure on eye movements. For example, it is unclear whether spontaneous gaze fixations after a tool is presented are attracted to either the tool head^6,7^, or to the handle^8^, or to the object’s center^9^. This apparent ambiguity may result from specific experimental paradigms as these studies used different methods for calculating gaze parameters. For instance both Myachykov et al. and Van der Linden et al.^8,9^ used gaze dwell times (time spent looking at a particular location) as their measure of visual attention, but yet used different methods for calculating these dwell times, potentially leading to the apparent discrepancy between their results (see^9^).

Most importantly, this previous research does not show whether tool ends (heads or handles) automatically affect oculomotor preparation in a way similar to how handles may facilitate preparing hand responses in certain context. Showing that saccades are primed by handles could suggest that the previously-described hand priming by tool handles results from automatic, effector-unspecific shifts of attention towards the handle. And conversely—if the saccades would be primed by tool heads, this could imply that attention mediates disparate sensorimotor programs for eyes and hands. In fact, some evidence suggests that visual attention is captured by the tool head^10,11^, but not by the handle, as probed using manual reaction times in attentional tasks. If this is the case, one would expect that eye saccades may be likewise primed towards the tool head, reflecting these putative covert shifts of attention. Interestingly, since both these previous studies^10,11^ used manual responses and did not measure saccadic reaction times, one cannot determine whether saccades towards the tool head are indeed executed faster than those towards tool handles.

Here, we scrutinized the effects of tool structure on planning and execution of eye movements and tested the idea whether the tool heads prime eye saccades in a similar way as handles prime hand responses. For this purpose, we used an attentional cueing task where subjects were instructed to make saccades in response to a color change of the central fixation dot. Unknowingly to the subjects, these saccades could be either congruent with the location of the tool head or the handle. We used a high-speed eyetracker to probe transient differences in saccadic reaction times that could be elusive at lower sampling speeds, such as ones used in previous research.

## Methods

### Subjects

Twenty-eight subjects (eight males) took part in the experiment. The participants were psychology students, naive to the experimental hypotheses and were compensated with bonus course credit for their participation. All subjects were right-handed, had normal or corrected-to-normal vision and provided written consent prior to participation. All experimental procedures were performed according to the Declaration of Helsinki and approved by the Ethics Board at the Faculty of Psychology and Educational Sciences of the University of Coimbra. Two subjects had to be excluded from the sample due to poor data quality (loss of eye position during the experiment). This sample size was higher than those used in previous literature investigating eye movements and attentional effects in tool perception (compare e.g.:^6,9,11^).

### Apparatus

All experiments were performed using Tobii TX300 video eye tracker connected to an experimental computer running Opensesame v. 3.1^12^. We used the in-built monitor, set to 100% brightness and 50% contrast. The display was running at 1920 × 1080 pix. resolution and 50 Hz refresh rate. Eye data were sampled at 300 Hz. We used a chin-rest to stabilize subjects’ head position at 67 cm visual distance.

### Stimuli

For most of the time subjects were supposed to fixate on a brown, central fixation dot (ca. 1 degree vis. angle). Black target crosses (1.3 deg. vis. angle) were positioned at 12 deg. vis. angle from the central dot.

We used a set of twenty-one every day graspable objects that had a handle and an active end (e.g. pliers), or no handle (a bowl). Tools with handle-head structure could be displayed either horizontally or at 45°–60° rotation according to their normal way of grasping (see Fig. 1D, Supplement 1 and Supplement 2). Critically, we balanced the numbers of handled tools in horizontal and oblique orientations altogether. Some items could be presented in both orientations (see Supplement 1 for details). The control (no-handle) objects had no oblique orientation. Some items were presented multiple times to balance repetitions of the main conditions. Some of the objects had multiple slightly different exemplars in order to provide more repetitions but reduce subjects’ familiarity. Object identities were not balanced with respect to the number of repetitions, and a few objects were present in just one orientation (see Supplement 1 for details on the objects used and their presentation). It is critical to note that this did not affect the balancing of our experimental conditions (see below).

**Figure﻿ 1:**
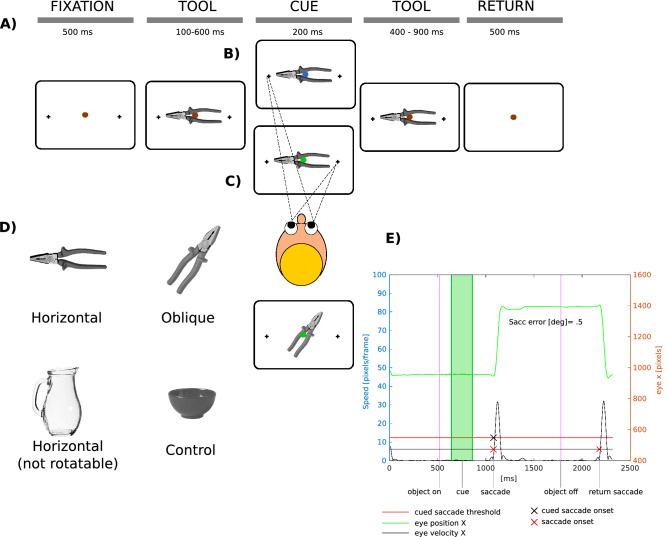
(**A**) Schematic depiction of a trial timeline for (**B**) head- and (**C**) handle-congruent saccades. See also Supplement 2 for an example timeline for oblique objects. (**D**) Example objects used in the experiment. (**E**) The saccade detection algorithm performance over an exemplary “green” trial timeline. The vertical bars denote epoch on/offsets (see labels), The green line is eye position X in screen coordinates plotted against time. Red crosses mark saccade onsets. Black cross denotes the onset of the saccade that was later taken for analysis. Figure created with Inkscape v. 0.92 (inkscape.org).

All objects were manually scaled to approximate their real-life size at 67 cm visual distance. Objects that were too big to be accurately represented on the screen (such as a basketball), were downscaled to a feasible size below 20 deg. visual angle. The objects with a handle were more elongated (average x:y ratio 15.2:5.1 deg. vis. angle; std x:y ratio 3.53:2.1 deg. vis. angle) than control objects (average x:y ratio 12.2:11 deg. vis. angle; std x:y ratio 5.6:6.7 deg. vis. angle).

### Task

We used an implicit precueing paradigm (Fig. 1A), in which subjects were instructed to detect a color change in a dot that indicated saccade left (blue dot), right (green dot) or no saccade (red). Subjects were instructed to focus on the color change detection and were told that tool images displayed in the background were not relevant for the task. Subjects were additionally instructed to fixate on the central dot whenever not saccading and withhold their blinking until the post-trial return phase. Each trial started with a 500 ms fixation. The tool object was flashed in the background and we manipulated the SOA between the image and color dot (tool image always appeared first). The SOA was 100, 200, 400 or 600 ms. In the “red” trials the SOA was always 400 ms. Saccades (left/right) were either congruent with handle (Fig. 1B) or the head (Fig. 1C), except for control items that did not have a handle/head. Importantly, the tool handles and heads were not overlapping with the target crosses, that means they did not cue the exact spatial target for the saccade. For oblique items the targets were likewise placed on the horizontal axis (see Supplement 2). Each tool image was always displayed for a total of 1000 ms. Afterwards, a blank screen with only the fixation dot was shown for 500 ms, instructing subjects to saccade back to the center of the screen.

The experiment consisted of 10 blocks, each with eighty-four trials. Per subject, we had 320 trials in which the saccade direction was congruent with tool’s head, 320 where the saccade was congruent with tool’s handle and 160 control trials. There were 40 repetitions of each “Tool-end” (handle/head/control) × SOA (100, 200, 400, 600) combination. There were 40 “red” (no saccade) trials in the whole experiment. These “red” trials were used for maintaining subjects’ attention and not included in the analysis.

As all object images were in grayscale, we displayed them on white background for visibility. We compensated for this and minimized subjects’ eye fatigue by having the experimental room brightly lit, and using frequent breaks between experimental blocks. No subject reported discomfort during the experiment.

### Analysis

All analyses were performed using custom routines coded in Matlab (Mathworks), based on raw eye data output obtained from Tobii SDK. Eye data were first low-pass filtered at 0.3 radians*pi/sample using a zero-phase filter with stopband attenuation of 60 dB applied to eye x and y position. We decided for this filter in order not to affect the signal’s temporal parameters. Then, the x and y eye positions were overwritten with the filtered data.

Eye blinks were detected on the basis of the loss of eye position over ten continuous samples, and 20 samples before and 20 samples after the blink were removed from the eye data in order to avoid spurious eye velocity distortions caused by eyelid closure/pupil size change. All data were then overwritten with blinks replaced by missing values (NaNs).

To calculate saccadic error, the saccadic landing points were averaged over nine samples after saccade end in order to compensate for the Gibbs phenomenon-related overshoot, resulting from the use of our filter (compare Fig. 1E). We rounded the saccadic error subject-average values to 0.03 deg (an equivalent of 1 pixel).

### Saccade detection

For saccade detection we used an eye-velocity-based algorithm. Eye velocities were calculated for x and y coordinates together. Then, two thresholds were applied: one for detecting all saccades (50 deg/s), and another one to detect big saccades of interest (100 deg/s), estimated according to saccadic amplitude/velocity ratio^13^. Saccadic onsets were detected if the velocity threshold was crossed over 3 consecutive samples. Saccadic offsets were detected when velocity dropped below threshold over 3 consecutive samples. See Fig. 1E for an example trial and visualized saccade detection algorithm performance.

## Results

**Figure 2:**
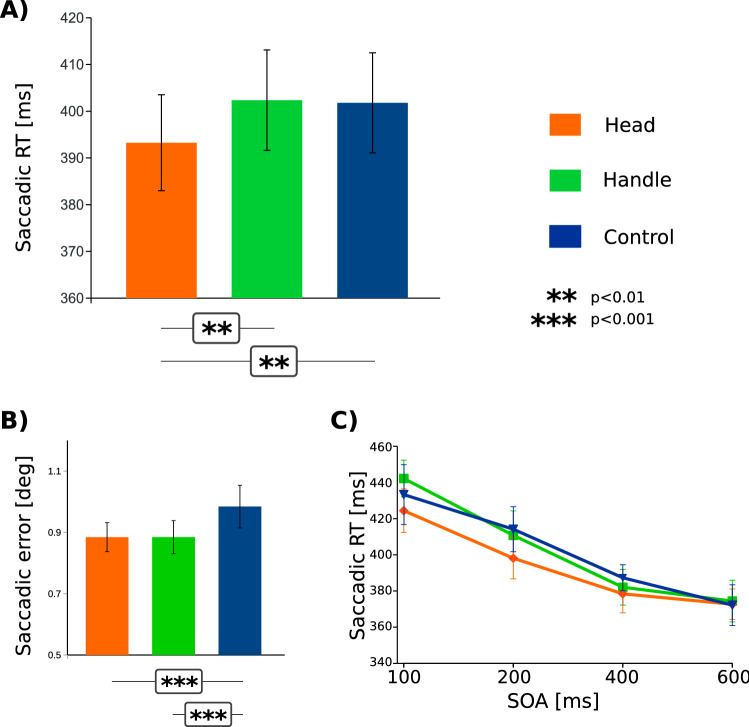
(**A**) Saccadic reaction times across conditions where saccades were congruent with the head (orange), the handle (green) and for control items (blue). Saccades executed towards the side congruent with head had significantly shorter latencies than those towards handle and control items. (**B**) There was no difference in the size of saccadic errors across both main experimental conditions (“Head” and “Handle). Both main conditions yielded saccades significantly more precise than in control condition. (**C**) Saccadic latencies for the main conditions plotted separately across all four SOAs. All error bars represent SEM. Figure created with Inkscape 0.92 (inkscape.org).

In order to scrutinize the effect of tool structure (handle vs. head) on saccadic reaction times, we performed a repeated-measures 3 × 4 ANOVA, with factors “Tool end” (three levels denoting congruency between tool side and saccade direction: “Head”, “Handle”, “Control”) and “SOA Timing” (Levels: “100”, “200”, “400”, “600” (ms)). This analysis showed a main effect of tool end (*F*(1.6, 40) = 6,97; *p* = 0.005 G-G corr., $\upeta _{{\text{G}}}^{2}$ = 0.005) and the relevant post-hoc t-test test uncovered significantly shorter saccadic reaction times for saccades directed towards the tool’s head (M = 393 ms; SEM = 10.3 ms) than for the tool handle (M = 402 ms; SEM = 10.8 ms): “Head” − “Handle”: *t*(25) = 3.33, *p* = 0.005, means diff. = 9.1 ms). Saccadic RTs towards tool heads were also shorter than for the control (no handle) items (M = 402 ms; SEM = 10.7 ms; “Head” − “Control”: *t*(25) = 3.127, *p* = 0.005). Figure 2A shows average saccadic reaction times for the “Tool end” conditions. There was no significant difference between saccades towards the tool handle and control items (“Handle” − “Control”: *t*(25) = 0.2, *p* = 0.98, means diff. =  − 0.56 ms). This difference was present in 21 out of our 26 subjects. There was a significant main effect of SOA timing (*F*(2.04, 51.05) = 59.3; *p* < 0.001 G–G corr.; $\upeta _{{\text{G}}}^{2}$ = 0.14; Fig. 2C), with reaction times shorter for longer SOAs (“100”: M = 433 ms; SEM = 12.3 ms; “200”: M = 408 ms; SEM = 12.0 ms, “400”: M = 383 ms; SEM = 8.64 ms, “600”: M = 373 ms; SEM = 10.2 ms). There was no interaction effect between the main factors (“Handle” × “SOA Timing”: *F*(2.84, 71.04) = 10.951; *p* = 0.461 G–G corr.; Fig. 2C).

Furthermore, we additionally investigated the potential interactions between tool structure, tool orientation and early and late attention. To this end, we performed the analysis of saccadic reaction times across all four SOAs between the tool and the saccade cue. We used repeated measures ANOVA with factors “Tool end” (Levels: “Handle” & “Head”), “SOA Timing” (Levels: “100”, “200”, “400”, “600” (ms)) and “[Tool] Orientation” (Levels: “Horizontal”, “Oblique”) As the control items had no oblique orientations, we did not include them in this analysis. This analysis again yielded significant effects of “Tool end” (F(1, 25) = 9.32; *p* = 0.005, $\upeta _{{\text{G}}}^{2}$ = 0.005) and “SOA Timing” (F(2.28, 56.99) = 92.75; *p* < 0.001 G–G corr., $\upeta _{{\text{G}}}^{2}$ = 0.14; Fig. 2B). There was neither the main effect of “Orientation” (F(1, 25) = 0.16, *p* = 0.7), nor “Tool End” versus “SOA Timing” interaction (F(2.54,63.53) = 1.7; *p* = 0.17 G–G corr.), nor “Tool-end” versus “SOA Timing” versus “Orientation” (F(2.05, 5126) = 1.62; *p* = 0.2 G–G corr.).

Lastly, we analyzed saccadic error sizes across the main “Tool end” conditions in order to scrutinize whether saccadic precision was affected by tool head/handle, which could suggest additional effects of spatial attention on our main conditions of interest (Fig. 2B). We used repeated measures ANOVA with three main levels: “Head”, “Handle”, “Control”. Although this analysis uncovered a significant effect of “Tool end” (*F*(1.35, 33.7) = 12.4; *p* < 0.001 G–G corr., $\upeta _{{\text{G}}}^{2}$ = 0.026), this effect was driven by the difference between both main conditions and the control condition, as uncovered by post-hoc tests (“Handle vs. Control”: *t*(25) =  − 4.6; *p* < 0.001; “Head vs. Control”: *t*(25) = -3.36; *p* < 0.003; means diff.: 0.09 deg. vis. angle). There was no difference between the “Head” and “Handle” conditions as the saccadic error sizes were identical (*t*(25) = 0; *p* = 1). Note that saccades in the main experimental conditions were only around 0.1 deg more precise than in the control condition. We furthermore performed a separate t-test to compare directly horizontal and oblique handled items. This comparison showed that for horizontal items saccades were more precise than for oblique items (*t*(25) = 3.16; *p* = 0.004; means diff.: 0.08 deg. vis. angle).

## Discussion

In this study, we used a pre-cueing paradigm to investigate whether and how the presence of a tool affects oculomotor preparation. We discovered that saccadic latencies are significantly shorter when saccadic direction is congruent with the tool’s head. Moreover, this effect was not due to “slowing down” of saccades towards the handle, as demonstrated by the lack of difference between handle and no-handle item conditions. Our results therefore show that human eye saccades are automatically primed by tool heads, but not tool handles. That is, tool heads automatically attract the eye saccade even if neither tool use nor recognition are part of task demands. Our results show that the eye is prepared for recognizing the tool’s head and its function and identity. That is, eyes are driven towards functional parts of manipulable objects, allowing to recognize their potential use^7,14^*.*

Using tools critically requires preparing and executing hand responses in order to grasp and manipulate a tool. This intimate link between tools and hands is reflected in the neural and cognitive overlap between how tools and hands are processed (e.g.^15–17^). Some experiments show that hand grasping might be automatically prepared in the presence of graspable tools. For example, tool handles (but not heads) were suggested to prime the speed of manual responses congruent with the handle side, for both button presses and grasps^18–22^. In fact, tool handles can  activate hand representations even during action observation^23^, suggesting that handles may be crucial for preparation of actions involving grasping a tool. This is especially vivid when a potential for action is recognized and a tool is placed in its relevant action context^24^. While these results are often considered compelling, they have been challenged by several lines of research showing that hand priming for handles might be heavily context-dependent and result from a specific experimental situation rather than reflect a general motor preparation of hands in the presence of tools^25–34^. Tipper et al. further showed that such putative hand priming might at least partially depend on whether the action affordance is implied by the task, for example when the objects are presented in their action-related states. The action states attenuate hand preparation even if it’s suppressed by other task demands, such as color cue detection (Tipper et al., 2006). Interestingly, and somewhat contrary to what Tipper et al. results showed for hand priming, in our experiment saccadic priming by tool heads was not suppressed by color detection task. This indicates that the priming of saccades by tool heads might be driven by distinct mechanisms than the putative priming of grasping by handles^26^.

Our finding that saccades are primed by tool heads seems complimentary to the previously described putative priming of hand responses in the presence of tool. Hand and eye movements in the presence of graspable tools are aimed at the different tool parts, and might be prepared at different points in time given the distinct ways of how these effectors interact with objects. Namely, the visual recognition of the tool’s identity and function by its distinctive head subsequently allows adjusting the grip posture for grasping the tool. This sequential preparation of eye and hand programs in tool recognition is reflected in distinct organization of the neural pathways processing visual information about tools. It has been shown that the recognition of manipulable objects is achieved through interactions between the dorsal and ventral streams within the tool processing network^1,3,17,35–38^. Handles are usually recognized by their elongated but coarse shape, which does not require the processing of fine spatial details, as grasping usually entails a coarse preshaping of the hand^39^. As a result, handle-related information is likely processed through the dorsal visual stream and magnocellular visual pathways under low spatial frequencies^3,39–42^, presumably for the purpose of quickly preparing hand posture for grasping^1^.

It seems clear why tool handles are vital for hand motor responses, however the relationship between eye movements and tool heads has been less straightforward. While some have suggested saccades are directed spontaneously towards tool handles^8^, our data shows that the saccades are by default prepared towards the tool’s head, in line with previous studies showing that tool heads attract visual attention^7,10,11^. Tool heads are critical for recognizing a tool’s identity, its function, and preparing for its use. Therefore, the rapid preparation of a saccade towards a tool head may be vital for the tool recognition process. After all, the main difference between an icepick and a screwdriver relates to fine details about their action tip.

Interestingly, these fine details are processed by the ventral stream and its parvocellular input^1,3^. The effect of tool heads on oculomotor behavior presented here may then be related with the fact that high-spatial frequencies are conveyed by the parvocellular visual pathway—it is likely that higher spatial frequency information drives visual attention towards the head of the tool^11^ and attracts the initial fixation^6^. That is, it allows for initial localization of the most distinct element of the tool—the head. Saccadic attentional preview^43^ may detect the head side of the tool and facilitate a saccade towards this distinctive element. By directing attention (and saccades) towards the head of the tool, the visual system is facilitating the extraction of important information for identifying a tool, its function, and other characteristics such as its weight distribution. This information can then percolate dorsal stream processing and influence representations of an object’s manner of manipulation^1,3,35,44,45^. This seems likely, as manipulable object recognition takes place through parallel processing, indicating an intensive exchange of information between the ventral and the dorsal stream components in tool recognition for action (e.g.^1,3,17,35–38,46,47^).

Curiously, we did not observe any substantial difference in saccadic reaction times depending on tool orientation. This might imply that the priming of saccades by tool’s head does not involve directing them to a specific spatial location. However, the lack of orientation effect could also result from an unbalanced proportion of distinct objects in different orientations, which could have confounded the hypothetical orientation effect and its related spatial priming. This remains to be further scrutinized. Interestingly, we also saw a small difference in precision (about 0.1 deg visual angle) between our tool objects, most of which were elongated, and control objects, most of which were round. Precision was higher for tools in horizontal orientation, consistent with saccade axis. This could suggest a further interplay between saccades and object elongation, a known factor determining the processing of graspable objects perhaps in the service of object manipulation (see e.g.^39,48^). However, for our study it is important to emphasize that there was no difference in saccadic RT’s between the handle-congruent and control conditions, showing that elongation per se does not affect saccadic priming in the way tool heads do.

One could suggest that the effect of shorter saccadic latencies in our study for head-congruent trials could result from typical Posner-like shifts of attention towards the side cued by the head. In such case, tools would be perceived as other stimuli conveying directional information, such as arrows. If that was true, we  could likewise observe the inhibition of return^49^ resulting in longer saccadic latencies in the handle-congruent condition (as the “cued” attention needs longer time to be redirected to the new stimulus). This was not the case and we did not find any difference between the handle and no-handle congruent conditions However, it is also worth to note that such transient, endogenous shifts of attention such as those evoked by faces (possibly similar to tools) do not necessarily evoke subsequent inhibition of return^50^, so we can not determine whether our results can be explained solely by global attention shifts.

Overall, it appears that tool handles and heads might be processed by neural circuits providing complimentary representations of handle- and tool-function related affordances. This distinct processing further implies distinct roles for eye and hand movement preparation in the presence of tools. The previous findings showed that hand actions such as grip preshaping, button presses etc. are primed by tool handles, suggesting unspecific motor preparation of grasping in the presence of handles. Our results, in turn, show that eyes are automatically primed towards the more distinctive and feature-rich tool’s head, potentially for the purpose of recognizing the tool’s distinct identity and function.

## Supplementary Information

Supplementary Information.
