# Supplementary material for: Tool heads prime saccades
Source: Sci Rep. 2021 Jun 7;11:11954. doi: 10.1038/s41598-021-91254-8 (PMC8184872; doi:10.1038/s41598-021-91254-8)
Supplement: Supplementary file 1 — Supplementary Information. [file 41598_2021_91254_MOESM1_ESM.pdf]

Tool heads prime saccades

Pilacinski A., De Haan S., Donato R., Almeida J.

Supplementary information

Supplement 1

**Item**	**Variants**	**Repetitions**	**Image orientations**			
			Oblique-Left	Oblique-Right	Horizontal-Left	Horizontal-Right
Bottle opener	1	32	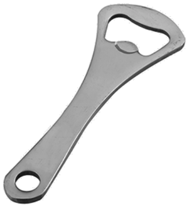	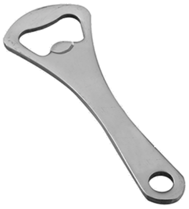		
Pliers	3	56	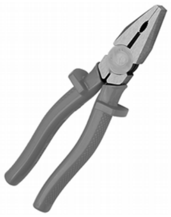	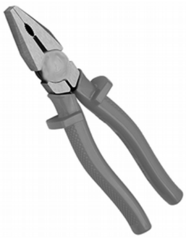	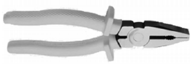	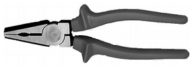
Hand mixer/beater	3	64	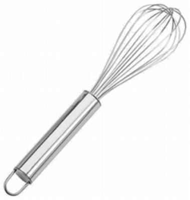	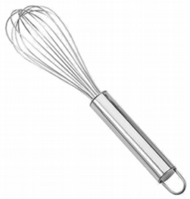	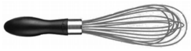	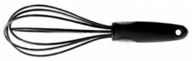
Pocket knife	1	16	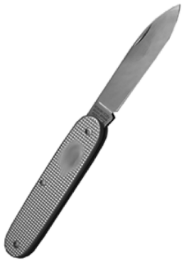	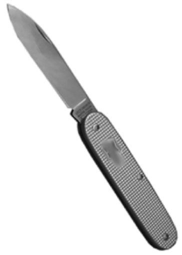		
Wrench	2	40	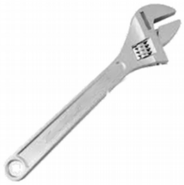	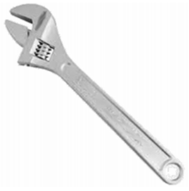	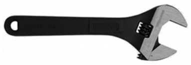	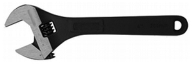

Peeler	4	48	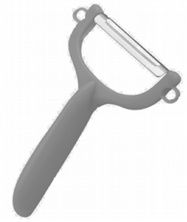	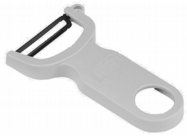	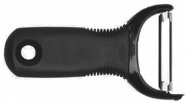	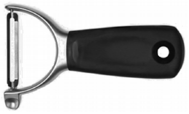
Plunger	2	48	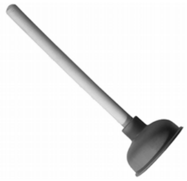	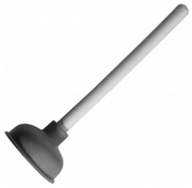	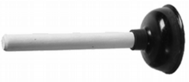	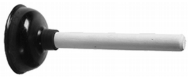
Hairbrush	3	40	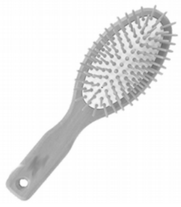	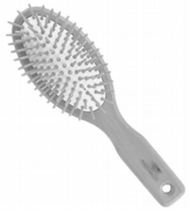	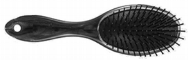	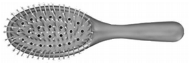
Toothbrush	3	48	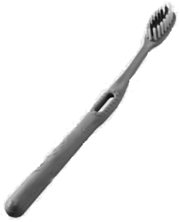	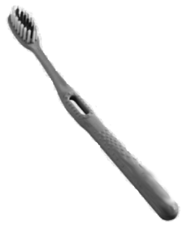	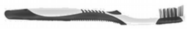	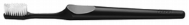
Knife	3	48	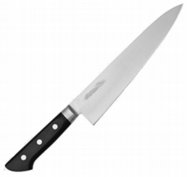	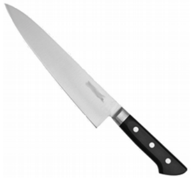	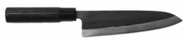	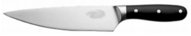
Bottle	1	16	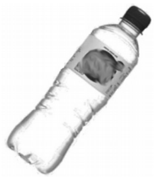	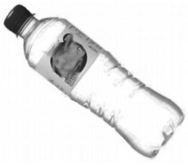		
Jug	1	8			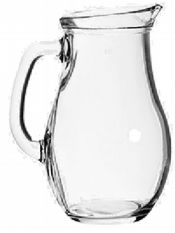	

Flashlight	1	16	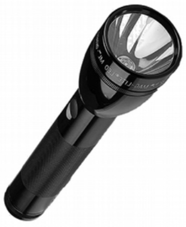	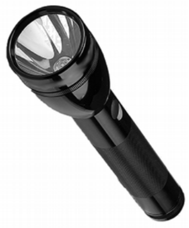		
Magnifier	2	40	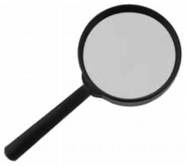	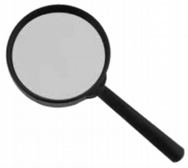	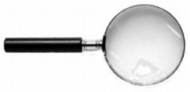	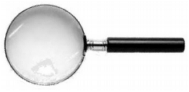
Electric shaver	2	24			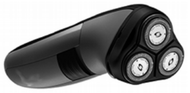	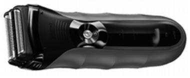
Hammer	3	40	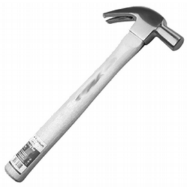	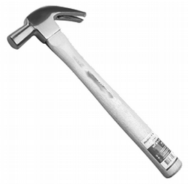	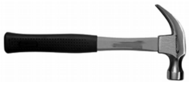	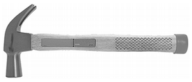
Nutcracker	2	24			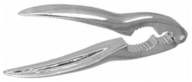	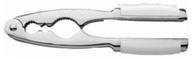
Racket	2	32	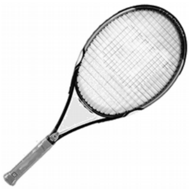	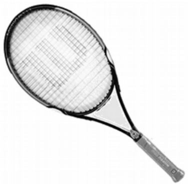	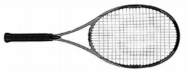	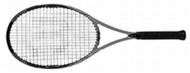
**Controls:**						
Basketball	1	80	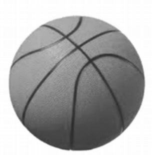			
Bowl	2	80	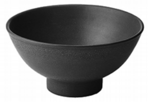			

Supplement 2

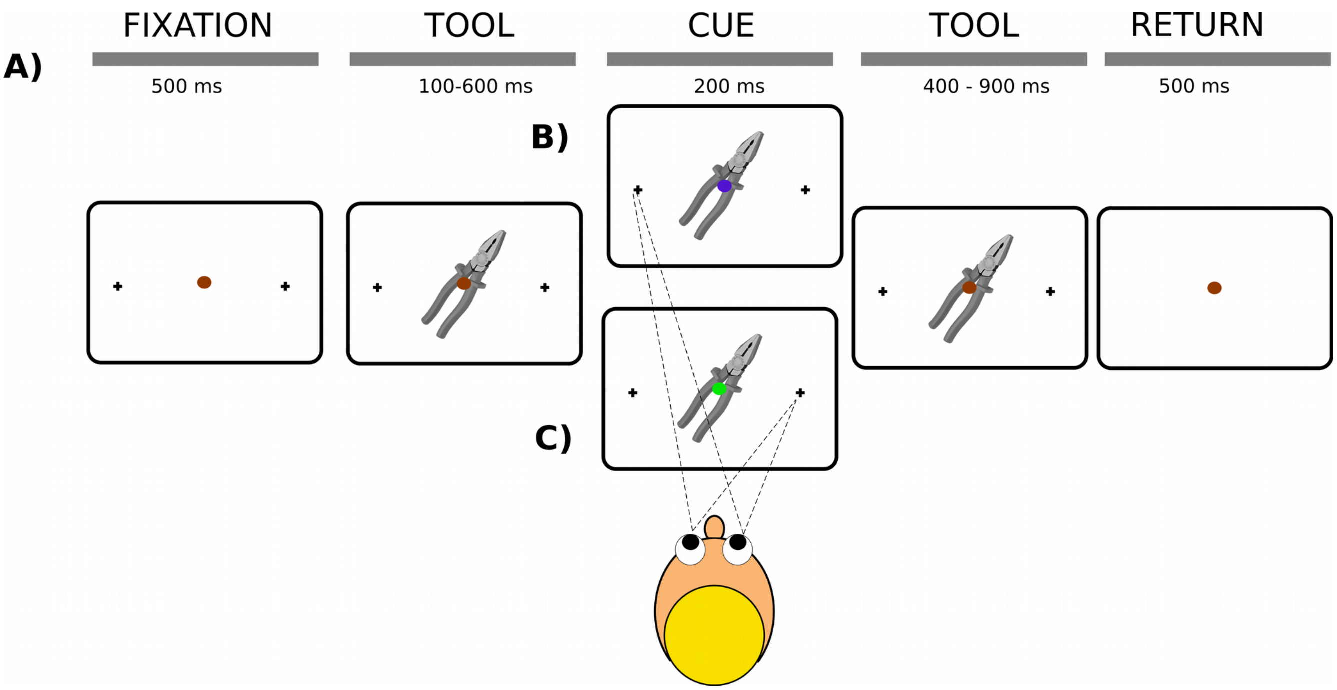

Schematic depiction of a trial timeline for B) head- and C) handle-congruent saccades for oblique items. Note the target crosses remain in the same plane as for horizontal and control items. Figure created with Inkscape 0.92 (inkscape.org).
